# Anti-metastatic Semi-synthetic Sulfated Maltotriose C-C Linked Dimers. Synthesis and Characterisation

**DOI:** 10.3390/molecules17089912

**Published:** 2012-08-17

**Authors:** Elena Vismara, Alessia Coletti, Antonio Valerio, AnnaMaria Naggi, Elena Urso, Giangiacomo Torri

**Affiliations:** 1 Department of Chemistry, Materials and Chemical Engineering “G. Natta”, Politecnico di Milano via Mancinelli 7, 20131 Milan, Italy; Email: alessia.coletti@chem.polimi.it (A.C.); antonio.valerio@chem.polimi.it (A.V.); 2 Scientific Institute of Chemistry and Biochemistry “G. Ronzoni”, via G. Colombo 81, 20133 Milan, Italy; Email: naggi@ronzoni.it (A.N.); mass_lab@ronzoni.it (E.U.); torri@ronzoni.it (G.T.)

**Keywords:** sulfated C/O maltotriosyl dimers, heparin, kink motif, NMR, metastasis

## Abstract

This manuscript describes the preparation and the spectroscopic characterisation of semi-synthetic sulfated maltotriose C-C linked dimers (SMTCs) where the natural C-O-C anomeric bond was substituted by one direct central C-C bond. This C-C bond induces conformation and flexibility changes with respect to the usual anomeric bond. SMTCs neutral precursors came from maltotriosyl bromide electroreduction through maltotriosyl radical intermediate dimerisation. The new C-C bond configuration, named for convenience α,α, α,β and β,β as the natural anomeric bond, dictated the statistic ratio formation of three diastereoisomers. They were separated by silica gel flash chromatography followed by semi preparative HPLC chromatography. Each diastereoisomer was exhaustively sulfated to afford the corresponding SMTCs. SMTCs were huge characterised by NMR spectroscopy which provided the sulfation degree, too. α,α and α,β were found quite homogeneous samples with a high degree of sulfation (85–95%). β,β appeared a non-homogeneous sample whose average sulfation degree was evaluated at around 78%. Mass spectroscopy experiments confirmed the sulfation degree range. Some considerations were proposed about SMTCs structure-biological properties.

## 1. Introduction

Carbohydrates have often pushed researchers to correlate biology and medicine topics to synthetic organic chemistry. In fact the multiple carbohydrate biological roles discovery was followed by huge efforts to modify natural structures for modulating their activities and to project non-natural carbohydrate-based compounds with new capabilities and applications. In advance, the great evolution of analytic and spectroscopic techniques enhanced isolation procedures and structural characterisations at such a high sophisticated level that was impossible to foresee even in a recent past.

Heparin, a natural polysaccharide which is bioactive in multiple directions, is a heterogeneous, polydispersed, highly sulfated glycosaminoglycan [1]. Heparin exhibits a number of biological activities including inhibition of thrombin generation [2], release of tissue factor pathway inhibitor from endothelial surfaces [3], modulation of growth factors receptor binding and activity [4] affecting angiogenesis [5], heparanase enzymatic activity [6], and selectin-mediated cell interactions [7].

Many of these activities relate heparin with cancer treatment, in particular with cancer metastasis control which remains the major obstacle in the treatment of cancer patients. Metastasis is a multiple-step process encompassing tumor cell release from primary sites, their survival in the circulation, and extravasation in distant tissues [8]. Metastasis is determined by the cellular origin, intrinsic features of the tumor, and the adhesive properties of tumor cells.

Developments of compounds that target heparanase are emerging as a promising approach for cancer therapy. Heparanase is an endoglycosidase that degrades heparan sulfate in the tumor microenvironment, cell surfaces, and vessel wall. An impressive number of papers has been published, as heparanase is up regulated in essentially all human tumors examined [9,10,11,12,13]. Moreover, inhibition of heparanase resulted in attenuation of metastasis in a number of animal models [14]. Since 1987 non-anticoagulant heparin species have also been found to inhibit heparanase [15]. Selectins are vascular cell adhesion molecules that mediate tumor cell interactions with platelets, leukocytes, and the vascular endothelium. The contribution of P-selectins to metastasis was identified in 2002 [16] and further detailed in 2007 [17]. Acting together heparanase and selectin facilitate tumor cell arrest, extravasation, and metastasis. Heparin has long been known to possess anti heparanase activity and to effectively inhibit P- and L-selectins. According to these activities, many studies have been done to provide heparins and heparin oligosaccharides as useful tools against cancer [18,19,20,21]. Some chemically modified heparin species were also reported to exert a beneficial effect in cancer patients, regardless of the mode of action [22].

However the use of heparin or low molecular weight heparins as anticancer agents is limited because of the risk of inducing adverse bleeding complications. This is the reason why explorations of the therapeutic potential of heparin mimetics have gone hand-in-hand with the emergence of understanding heparin potentiality as anti-metastatic drug [23]. Actually, it should be emphasised that the real bioactive moieties of natural polysaccharides are specific or unspecific oligosaccharides of more than four sugar units and that, due to their small molecular size, oligosaccharides show a larger bioavailability then their polymeric precursors [24]. As a result, large efforts have been addressed to isolating sequences of natural oligosaccharides, which possess a significant biological function. Among them, PI-88 is the sulfated form of phosphomannopentaose and phosphomannotetraose oligosaccharide mixture derived from yeast [25]. PI-88 has been found to act as a potent antiangiogenic, antitumor and antimetastatic agent. It inhibits the heparan sulfate-degrading enzyme heparanase, exhibits antiangiogenic activity and has anticoagulant properties mediated by heparin cofactor II [26,27]. Maltohexaose was another oligosaccharide whose sulfated forms have been found efficient inhibitors of tumour growth and metastasis [28,29].

**Figure 1:**
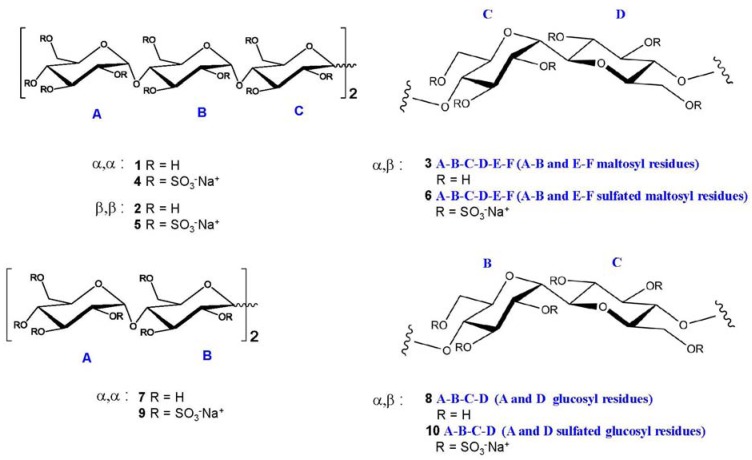
Structures of the maltotriose and maltose C-C linked dimers **1**–**3** and **7**,**8** and of their sulfated forms (SMTCs) **4**–**6** and **9**,**10**.

Starting from maltohexaose we have begun focusing our attention on the malto-oligosaccharide skeleton. In particular we moved to building up mixed O/C maltohexaose that are maltotriose C-C linked dimers **1**–**3**, see Figure 1, and to check whether or not their sulfated forms **4**–**6** would mimic heparin.

We have named them for convenience Sulfated Maltotriose C-C linked dimers (SMTCs). They are characterised by the presence of an interglycosidic C-C bond whose configurations lead to conformations different from the usual anomeric bonds. As a consequence SMTC chains appear in many aspects different from the regular maltohexaose chain. As shown in Figure 1, we also prepared maltose C-C linked dimers **7**,**8** and their sulfated forms **9**,**10**.

It is well-known that sugar conformations are crucial for biological interactions. In particular heparin has been found to present a peculiar “kink motif”, mainly due to the periodic presence of different uronic acid along the sugar unit sequence [30]. This kink conformation was found to make heparin chain suitable to interact with different protein binding sites. Noteworthy the “kink motif” has been identified by using heparin oligosaccharides. We already showed that the preferred C-C α,β gauche conformation of **3** presents a kink [31]. As a consequence **6** could mimic heparin chain even much better than the linear sulfated maltohexaose.

**Scheme 1:**
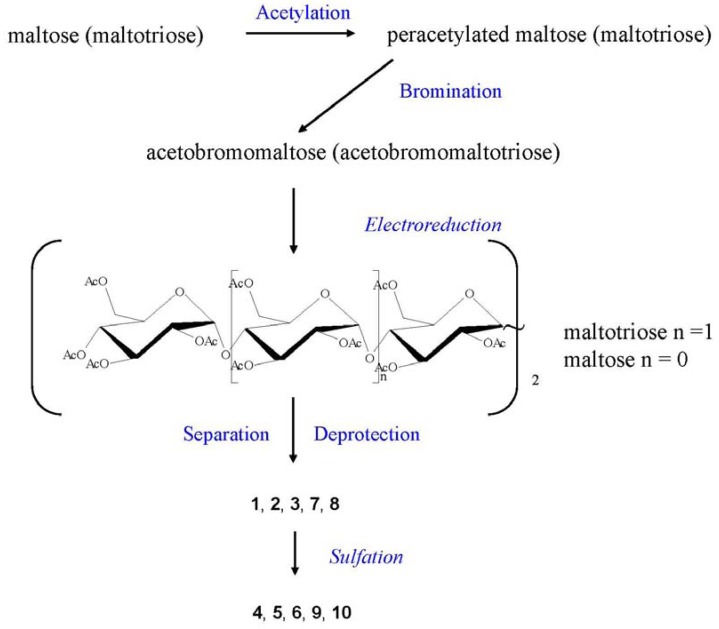
Summary of **4**–**6** and **9**,**10** preparation from commercially available maltotriose and maltose.

In advance, SMTCs belong to the C-glycoside family, which are less vulnerable to metabolic processing than their O-analogues and for this reason have been studied as drug candidates and inhibitors of carbohydrate-processing enzymes [32]. Eventually, the presence of the direct interglycosidic C-C bond makes SMTCs conformation rigid. This is an important feature as the conformational flexibility of oligosaccharides is critical in determining their binding to protein and consequently their bioactivity [33,34]. Scheme 1 summarises all the synthetic steps that starting from maltotriose and maltose afforded sulfated maltotriose C-C linked dimers **4**–**6** and sulfated maltose C-C linked dimers **9**,**10**.

Routine procedures have been used to prepare acetobromomaltotriose and acetobromomaltose. Acetobromomaltotriose and acetobromomaltose electroreduction on silver cathode, a well-established technique providing a mild, clean and one-pot method to double sugar units via the formation of stable interglycosidic C-C bonds by dimerization on anomeric radicals, afforded the **1**–**3** and **7**,**8** peracetylated precursors, respectively [31]. The following steps afforded **1**–**3** and **7**,**8**. We have defined **1**–**3** as maltotriose C-C linked dimers and **7**,**8** as maltose C-C linked dimers, respectively, just to underline the novelty of the structure and the originality of the reaction pathway.

**1**–**3** were sulfated to afford SMTCs **4**–**6**. **4** and **5** were found to attenuate metastasis by inhibition of P-selectin and heparanase [35]. Their activity against different tumors depends on the C-C configuration and was put in relationship with their heparanase and selectin inhibitory properties [36].

In this paper we describe how to isolate peracetylated forms of **1**–**3** on a 50 mg scale and their crucial following sulfation process to afford **4**–**6**. **4**–**6** were submitted to exhaustive NMR characterisation which provided also their sulfation degree that was further confirmed by mass spectroscopy. **9**,**10** were prepared from **7**,**8** and characterised, too. Their spectra have been usefully compared with those of **4**–**6**. At the moment **9** and **10** appear useless from a biological point of view as they did not show any heparanase inhibition activity.

## 2. Results and Discussion

The first step to achieve SMTCs was the acetobromomaltotriose electrochemical reduction [31]. After the cathode solution workup, the peracetylated diastereoisomers mixture was separated from the raw reaction mixture by silica gel flash chromatography. As detailed in the experimental section, it was impossible to fully separate the three components simply by flash chromatography, due to the quite similar *R_f_*. This separation was performed by HPLC semi-preparative chromatography which is actually crucial for our purpose to get pure compounds. Their availability is one of the most important features that often makes the difference between synthesis and natural product isolation and allows attaining precise correlation structure-properties. Once obtained pure peracetylated diastereoisomers, it was routine chemistry to deprotect them to afford SMTCs precursors **1**–**3** and **7**,**8** [31]. The final crucial sulfation step which afforded SMTCs **4**–**6** and **9**,**10** was performed in pyridine by means of SO_3_–pyridine complex.

Sulfate esters are now recognised as modulators of a number of important physiological and pathological processes [37]. Regarding highly negative charged polysaccharides, in the class of unique molecules generated by biosynthetic sulfation, the specific sequences present in polymeric heparin/heparan sulfate is nature’s engineering feat. SMTCs were thought as heparin mimetics and they were expected to have multiples biological potentialities. SMTCs can be considered in between small molecules and polymers. As concern their synthesis, the aim was to reach for each diastereoisomer as highest sulfation degree as possible. The idea was that fully sulfated structures can be preliminary tested for their inhibition properties. If they are active the following challenge is to study their specific binding with the inhibition site to discriminate what sulfates are really essential. Thus we decided to start with unique sulfated compounds. The major challenge was driving the reaction to completion to sulfate all available OH functional groups. Generally speaking, as the number of alcoholic groups increase on a small scaffold, sulfation becomes progressively difficult because of anionic crowding, resulting in numerous partially sulfated side-products. In advance, nearly all sulfated molecules are water soluble, which makes them difficult to isolate in highly pure form; a common problem is the presence of inorganic salts, the proportion of which is usually higher at small synthetic scales.

Reference [37] reports an exhaustively overview of sulfation protocols, *i.e.*, sulfation using sulfuric acid, dicyclohexylcarbodiimide-mediated sulfation and sulfur trioxide amine complexes. Sulfation using sulfur trioxide–amine complexes of SO_3_ with organic bases including pyridine (Py), trimethylamine (NMe_3_), and triethylamine (NEt_3_), or amides such as DMF have typically found extensive usage. NMe_3_ and NEt_3_ complexes with SO_3_ appear to be well suited for sulfation of alcoholic groups present in carbohydrates, steroids, and aliphatic or alicyclic scaffolds.

SO_3_–Py complex has been most often used for sulfation of carbohydrate scaffolds from mono-, oligosaccharides to polysaccharides by modulating the sulfation degree (DS), too. For example there is an anti-ulcer agent, sucrose octasulfate prepared by sucrose total sulfation through SO_3_–pyridine complex [38]. DS of sulfated galacto-oligosaccharides (from 3 to 7 sugar units), maltohexaose and heptaose studied for their effects on angiogenesis and prepared also through SO_3_–pyridine complex varied from 35 to 64% [28,39]. Synthetic heparin oligosaccharides have been obtained by SO_3_–pyridine complex [40]. For the synthesis of galactomannan sulfates, an attempt has been made to correlate the DS with the temperature by using SO_3_–Py complex in comparison with chlorosulfonic acid in an organic alkali. Preliminary analysis of the ^13^C-NMR spectra of the guar derivative has also been reported showing that the sulfate groups primarily substitute the hydroxy groups at C6 of the galactopyranose residues [41]. Recently, a microwave-based protocol with SO_3_–NEt_3_ complex has been developed to enhance the rate of sulfation of phenolic structures, especially those with multiple phenolic groups [42]. It is also interesting to cite that the same microwave-assisted sulfation of heparin oligosaccharides has been recently described to improve yields, although the starting oligosaccharides have only one or two position to sulfate for each sugar units [43]. In contrast to the above microwave-assisted sulfation, which can be categorised as high temperature, base catalysed reaction, Krylov et al. have recently reported low temperature, acid-catalysed sulfation reaction by SO_3_ complexes [44,45].

According to the above background we chose the more common and experienced protocol of SO_3_–Py complex in Py at 80 °C. Noteworthy, both microwave-assisted and acid-catalysed sulfation offer in our opinion very interesting perspectives for our further achievements.

As detailed below high sulfation in pyridine by means of SO_3_–pyridine complex was successful with **4** and **6**. **5** was much less sulfated and was a mixture of different sulfation pattern. We can argue that the C-C bond configuration of **1**–**3** is an important parameter for their reactivity toward sulfation reaction. **1** and **3** have quite the same behavior while **2** is very different.

On the other hand the biological activities of **4** and **6** seem to depend on C-C configuration. In fact **4** and **6** that had a quite similar degree of sulfation showed some differences in biological activities as they both are heparanase inhibitors, **6** better than **4**, but only **6** inhibits selectin [29]. The different behavior could be put in relationship with the preferred conformation associated with C-C bond configuration. Compound **4** is weakly bent due to its α,α *anti* conformation, while **6** has a huge bend due to the α,β *gauche* conformation. Concerning conformation and biological activities, **6** appears quite similar to heparin. Eventually preliminary data showed that **5** acts as very low heparanase and selectin inhibitor, this could be put in relationship both with the low degree of sulfation and with a linear structure suggested by the β,β C-C configuration.

### 2.1. Synthesis of Sulfated Maltotriose C-C Linked Dimers ***4**–**6*** and Sulfated Maltose C-C Linked Dimers ***9**,**10***

Sulfation procedure was the same for all the samples. In order to reach high sulfation degree the procedure was repeated two or three times. All the α,α and α,β diastereoisomers afforded homogeneous high sulfation degree samples. β,β diastereoisomer sulfation was much more difficult to manage. There was an increase between the first and the second treatment but the third one was ineffective. Thus **5** is a mixture of differently sulfated compounds.

### 2.2. NMR Characterisation of Sulfated Maltotriose C-C Linked Dimers ***4**–**6***

Generally, carbohydrate sulfation induces a significant low field shift of signals in NMR spectra of the proton and the carbon bearing the sulfate ester with respect to the same signals in the non-sulfated forms. Accordingly, the ^1^H- and ^13^C-NMR spectrometry (1D-NMR) are rightly considered an informative analytical approach, while signals are dispersed in reason of the both shifts by combining the two techniques in the heteronuclear ^1^H-^13^C bidimensional spectroscopy (HSQC NMR).

**Figure 2:**
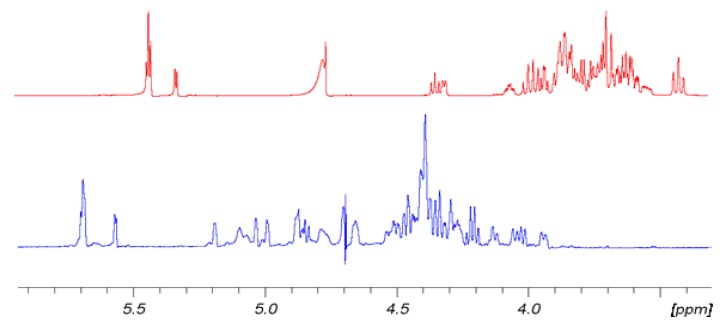
Comparison of ^1^H-NMR spectra of **6** (*blue*) at T = 303 K and its hydroxyl precursor **3** (*red*) at 298 K. 500 MHz in D_2_O.

The discussion of NMR characterisation of **4**–**6** deserves a particular attention. Figure 2 shows for convenience the shift effect on proton spectra of sample **6** with respect to the non-sulfated form sample **3**. The trend is the same for **5** with respect to **2** and for **4** with respect to **1**. It is evident how the sulfation process modifies the proton signal distribution. **6** shows very low signals in the region between 3.3 and 3.9 ppm where the protons of sample **3** resonate, indicating that non-sulfated structures are present only in trace. Signals assignment was done by using traditional bidimensional NMR techniques as COSY, TOCSY, HSQC, DEPT-HSQC and HMBC. In particular, HMBC was used to assign NMR signals to a specific ring of each oligosaccharide. Results are reported in Table 1,Table 2,Table 3. For convenience, it is anticipated here that in sample **5** two main differently sulfated structures were identified, see Table 3 for assignment.

**Table 1:** ^1^H and ^13^C NMR assignment of **4**. In bracket: *J_1_* and *J_2_* values, in Hz. 500 MHz, D_2_O. Capital letters indicate the name of the ring, as shown in Figure 1. Values expressed as ppm or (Hz).

H	A		B		C	
	^1^H (J)	^13^C	^1^H (J)	^13^C	^1^H (J)	^13^C
**1**	5.59 (d, 2.9)	96.6	5.48 (d, 3.4)	97.8	4.33 (d, 1.0)	69.9
**2**	4.75 (dd, 2.9, 5.3)	75.1	4.41 (dd, 3.1, 7.4)	76.3	5.01 (dd, 4.1)	74.0
**3**	5.04 (dd, 4.4)	76.9	4.87 (dd, 8.0, 9.2)	79.9	5.01 (dd, 2.1)	72.7
**4**	4.58 (dd, 3.8, 7.0)	75.3	4.10 (dd, 8.3, 18.6)	74.7	4.17 (dd, 2.5)	72.4
**5**	4.21 (ddd, 3.6, 7.1)	73.9	4.06 (ddd, 1.5, 2.1, 7.2)	72.3	4.42 (ddd, 3.3, 8.5)	77.1
**6**	4.38–4.33 (dd, 3.4, 10.8)	68.7	4.34–4.31 (dd, 3.4, 10.6)	68.7	4.27; 4.48 (dd, 4.9, 11.3)	67.6

**Table 2:** ^1^H and ^13^C-NMR assignment of **6**. Bracket: *J_1_* and *J_2_* values, in Hz. 500 MHz, D_2_O. Values expressed as ppm or (Hz).

H	A		B		C		D		E		F	
	^1^H (J)	^13^C	^1^H (J)	^13^C	^1^H (J)	^13^C	^1^H(J)	^13^C	^1^H (J)	^13^C	^1^H (J)	^13^C
**1**	5.67	96.2	5.69	97	4	71.5	4.47	78.8	5.56	97.4	5.67	96.2
	(d, 9.5)		(d)		(d, 9.5)		(m)		(d, 3.5)		(d)	
**2**	4.77	75.4	4.51	77.1	4.86	72.9	5.18	75.7	4.49	77.1	4.78	75.4
	(m)		(dd)		(t)		(nd)		(dd)		(m)	
**3**	5.07	77.1	4.83	80	5.02	74.2	4.98	78.2	4.86	80	5.09	77.1
	(d, 3.0)		(t)		(d, 3.0)		(d)		(t)		(d, 3.0)	
**4**	4.66	75.3	4.19	74.1	4.28	72.5	4.33	75.3	4.21	74.1	4.65	75.3
	(dd)		(dd)		(m)		(m)		(dd)		(dd)	
**5**	4.25	73.4	4.11	72	4.39	76.7	3.93	76.5	4.03	72	4.255	73.4
	(q)		(t)		(m)		(dt)		(t)		(q)	
**6**	4.45–4.36	69.5–68.0	4.39–4.35	69.5–68.0	4.47–4.35	69.5–68.0	4.32	70.2	4.376	69.5–68.0	4.43–4.38	69.5–68.0
	(m)		(m)		(m)		(m)		(m)		(m)	

**Table 3:** ^1^H and ^13^C-NMR assignment of **5** *.

H	A		B		C	
	^1^H (J)	^13^C	^1^H (J)	^13^C	^1^H (J)	^13^C
**1**	5.63 (d, 3.5)	96.5	5.56 (d, 3.7)	97.1	3.93 (d, 8.6)	76.8
**2**	4.52	76.0	4.82	80.0	4.64	76.1
**3**	4.81	77.8	5.04	78.0	4.07	78.8
**4**	4.51	78.7	4.21	72.6	3.77	79.2
**5**	N.A.	N.A.	4.60	75.5	4.32	78.5
**6**	4.36	70.5–68.4	4.39; 4.32	70.5–68.4	4.38	70.5–68.4
**H**	**a**		**b**		**c**	
	**^1^H (J)**	**^13^C**	**^1^H (J)**	**^13^C**	**^1^H (J)**	**^13^C**
**1**	5.76 (d, 3.8)	99.2	5.60 (d, 3.7)	96.5	3.66 (?)	78.5 (?)
**2**	4.41	77.8	4.72	79.9	3.88 (?)	78.6 (?)
**3**	4.81	79.9	5.02	77.2	4.01 (?)	78.9 (?)
**4**	4.09	74.6	4.19	73.4	3.77 (?)	79.2 (?)
**5**	4.16	72.2	4.59	75.4	N.A.	N.A.
**6**	4.35	70.5–68.4	4.39-4.32	70.5–68.4	N.A.	70.5–68.4 (?)

* Rings of the major product are indicated by capital letters (**A**-**B**-**C**) whilst small letters (**a**-**b**-**c**) are used for the minor one. N.A. stands for not assigned. 600 MHz, D_2_O. Values expressed as ppm or (Hz). Assignments of signals marked with question mark (?) are uncertain.

**Figure 3:**
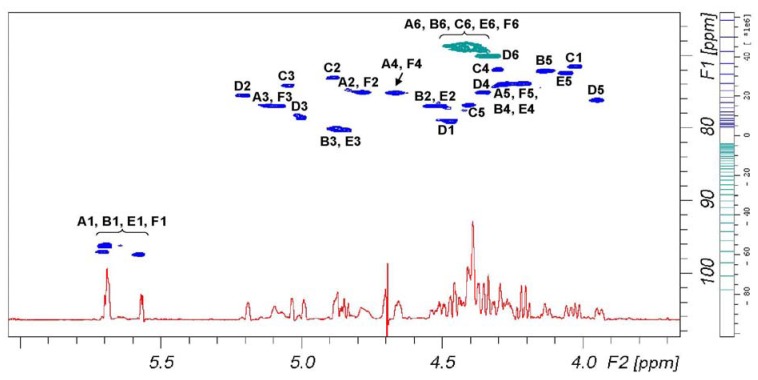
1D ^1^H-NMR (*red*) and ^1^H-^13^C DEPT-HSQC NMR spectra (positive phase (*blue*) and negative phase (*green*), respectively secondary and primary carbons) of **6**.

Figure 3 reports signal dispersion in the ^1^H-^13^C DEPT-HSQC spectrum of **6** that discriminates between primary and secondary carbons. According to Table 2, signals of secondary alcohol involved in the sulfation are comprise in a plane area defined by 4.44 and 5.25 ppm on proton axis and 72.5 and 80.7 ppm on carbon axis, while signals comprise between 4.50–4.28 and 68.0–70.0 ppm belong to primary alcohols. According to these assignments, we can see that primary hydroxyl groups are fully sulfated.

### 2.3. Evaluation of Sulfation Degree of ***4**–**6***

Generally speaking, due to the well-known reactivity of hydroxyl groups of sugar units, sulfation likely occurs at first on the primary alcohols, being the most reactive positions, as well as at the non-reducing end of hexamer structures. However, since the various positions of the glucose units reacts differently and the different structure of diastereoisomers seems to play a role, evaluation of the sulfation degree of each compound becomes necessary.

As stated by NMR theory, quantitative evaluation is possible by proton spectra. Because of 1D spectral signal overlapping only an average sulfation degree relative to H-2, H-3 of all rings could be calculated allowing, anyway, to compare the different compounds and to discriminate batch to batch preparations. Furthermore, the signal dispersion given by HSQC spectra is helpful to evaluate the sulfation pattern. In fact, if defined criteria of spectral measurements [46] are followed, each signal corresponding to single sulfated positions should be quantified. We found that for D ring the H-3 signal volume is not proportional to the other signal volume. So, the following approach was used to give a complete sulfation degree and some information relative to the sulfation pattern of the different diastereoisomers.

At first, the absence of ^13^C signals, in the range of 61.0–67.0 ppm in HSQC spectra of α,α, α,β and β,β diastereoisomers, attributable to the presence of primary hydroxyl groups, allows us to assume that such positions are totally sulfated, being the limit of detection of the technique better than 2%. Therefore, their 6-O-sulfation degree is around 100%, as already mentioned above. The ^1^H-NMR spectra of α,α and α,β sulfated diastereoisomers were considered, using the four regular anomeric signals integral value as calibration area. On the base of the NMR assignment, the integral of all H-2, H-3, H-4 non-reducing sites bearing a sulfate were evaluated.

The chemical shift range from 4.55 to 5.25 ppm could be used to measure the content of 12 over 20 possible sulfate residues, being signals of H-2 of sulfated B and E rings partially overlapping other signals. Such evaluation requires to set the residual H-O-D solvent signal in a free signal area of the spectrum, as Figure 3 shows, and to exclude it from integral area. The presence of residual solvent signals, suppressed by selective proton decoupling, could induce an underestimation of the two vicinal signals.

To consider the contribution to the DS of the two remaining H-2 signals of the B and E ring, the volume integral of the signals was measured in the HSQC spectra recorded according to Guerrini *et al.* [46] and compared to the volume area of the four anomeric signals used as calibrators. The percentage of recovery was confirmed fitting data also with other spectrum signals, in between the signal of two H-5 and two H-4 of rings, A, F, B and E. The average degree of sulfation is obtained with a good level of approximation adding up the three different measured values.

It is to note that intermediate sulfation degree gives complex spectra due to sequence effects. In such a case, spectra usually are affected by small change in chemical shifts, increment of number of signals and signals broadening giving a more signal crowded spectrum. It makes more complex the analysis of HSQC spectra. But being that the sulfate signal distribution remains inside the previous described area, the evaluation of an average sulfation degree is possible by 1D proton spectra that in the case become more potent than the 2D technique. Therefore the quantitative approach based on both 1D and 2D heteronuclear spectroscopy was preferred to the use of the single 2D one.

**Figure 4:**
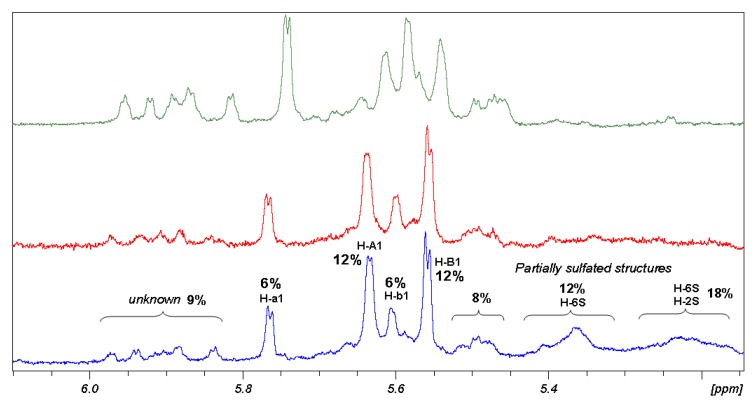
Comparison of ^1^H-NMR regions of anomeric protons of **5**, after one (*green*), two (*red*) and three (*blue*) sulfation reactions.

This is the case of β,β diasteroisomer. The range of chemical shift between 5.15 and 6.00 ppm is useful to analyse the composition of the β,β sample. The anomeric signals of proton spectrum compared with their sulfation intermediate (Figure 4) show the progressive decrease of heterogeneity of the sample without reaching a final homogeneity. Groups of signals have been identified using different correlation techniques. As a result, β,β sample is composed of two main structures having a well-defined NMR pattern, which assignment was reported in Table 3. Those structures represent more than 70% of the compounds and their ratio is 1:2. The remaining of the sample could not be studied because of structural polydispersion but in any case some 2D signal correlation allows to establish presence of signals of H-6 sulfated β,β hexamer (12%), H-6 and H-2 sulfated β,β hexamer (18%) and 20% of unknown structures of the β,β diastereoisomer.

The procedure used to calculate sulfation degree of maltotriose dimers interpreting 1D-NMR data is described in detail as follows. In the 5.80–5.35 ppm range of ^1^H-NMR only signals of anomeric protons of sulfated molecules are present, therefore the integral value divided for the numbers of protons resonating in that region was taken as the unit. The ratio between the integral of each region and the unit value, multiplied for the number of protons responsible for the signals, gives the degree of sulfation of functional groups resonating in that region, according to summarised formula (1):



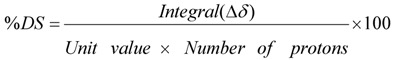
(1)


Due to the complex NMR profiles, only clear assignment regions were selected to calculate the sulfation degree average value, applying formula (2):



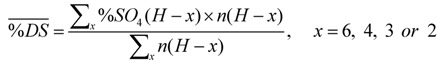
(2)


where *n* is the number of protons and *x* indicates the number of the positions.

Results obtained, collected in Table 4, show that **5** is the less sulfated structure, even after three sulfation steps (Figure 4).

**Table 4:** Sulfation degree obtained for α,α **4**, α,β **6** and β,β **5** samples of C-C linked maltotriose dimers. Value for **5** is given as an average between major and minor products.

Diastereoisomer	Sulfation degree
α,β **4**	>95%
α,β **6**	84%
α,β **5**	78%

Percentage of sulfation obtained for α,α and α,β diastereoisomers means that likely 17–19 hydroxyl groups are sulfated among the 20 possible positions. β,β maltotriose dimer appears to have only 15 sulfated positions. To note, positions H-6 are always considered totally sulfated. This is an average value; it does not correspond to the DS of the two defined structures, being those chemical shifts aligned to that of the α,α and α,β diastereoisomers. In fact, assignment of ^1^H- and ^13^C-NMR spectra of β,β diastereoisomers reveal that the high degree of sulfation and the difference between the two structures are due to the H-4 site of ring A that in the major product is sulfated, having a signal at 4.51 ppm, while the species present in minor quantity bear a hydroxyl group in the same site. A second difference seems to be linked to site H-2 of C rings but it remain doubtful because of uncertain assignments of the minor structure.

**Figure 5:**
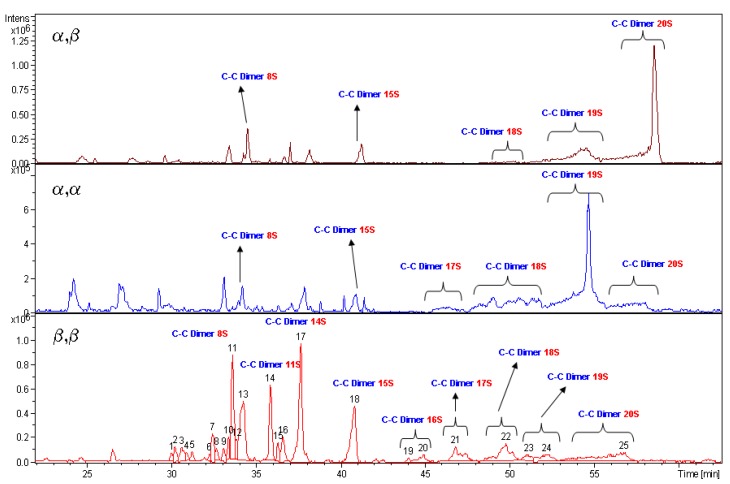
LC-MS analyses of α,β **6**, α,α **4** and β,β **5** compounds. Labels indicate the number of sulfate groups for detected species.

The high value of DS obtained from the above discussed NMR data are supported by LC-MS measurements. The species bearing 20 sulfate groups have been detected by mass spectroscopy for each diastereoisomer. The spectrum profiles, run under same liquid chromatography conditions, are reported in Figure 5. Unfortunately the MS technique is not a quantitative one. The intensity of mass signals depends from the molecular mass, so the higher is the degree of sulfation of a molecule the lower is the signal intensity. In fact, **5** having a more heterogeneous pattern in the region of low molecular weight species show high signals. **4** and **6** diastereoisomers are characterised by the presence of low signals dispersion. It is relevant to note that, in spite of the previous statement, **4** and **5** show, each one, a high intensity and prevalent signal, respectively for structures bearing 19 and 20 sulfate residues.

### 2.4. NMR Characterisation of Sulfated Maltose C-C Linked Dimers ***9**,**10**.* Comparison with ***4**–**6***

As evidenced by their NMR characterisation reported in Table 5 and Table 6, **9** and **10** were obtained at an almost total sulfation degree.

To evaluate the influence of the chain length, ^1^H-NMR chemical shift δ values for each position of **9**,**10** and **4**–**6** have been compared. Their difference was calculated for each position according to Equation 3, and summarised in Table 7 and Table 8.



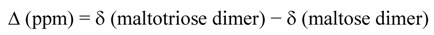
(3)


**Table 5:** ^1^H and ^13^C-NMR assignment of **9**.

H	A		B	
	^1^H (J)	^13^C	^1^H	^13^C
**1**	5.541 (3.4)	97.9	4.376 (1.6)	70.3
**2**	4.445 (9.8)	77.2	5.054 (nd)	72.8
**3**	4.848 (8.5)	78.1	5.117 (3.4)	74.2
**4**	4.501 (9.8)	76.7	4.241 (nd)	73.6
**5**	4.125 (m)	72.2	4.472 (m)	76.1
**6**	4.387 (3.5;11)	67.9	4.386 (dd)	69.1
**6**	4.439 (3;11)	-	4.437 (dd)	-

**Table 6:** ^1^H and ^13^C-NMR assignment of **10**.

H	A		B		C		D	
	^1^H (J)	^13^C	^1^H (J)	^13^C	^1^H (J)	^13^C	^1^H (J)	^13^C
**1**	5.668 (3.8)	97.0	4.019 (2.3;9.7)	72.0	4.441 (9.8;1)	78.8	5.555 (3.4)	97.6
**2**	4.457 (dd)	77.1	4.884 (2.6;2.4)	73.3	5.189 (m?)	75.7	4.447 (dd)	77.2
**3**	4.732 (dd)	78.3	5.109 (1.3;3.3)	74.6	5.026 (1;3.2)	78.1	4.810 (dd)	78.2
**4**	4.496 (dd)	76.7	4.275 (dd)	73.5	4.353 (9)	75.5	4.532 (dd)	76.6
**5**	4.090	72.0	4.368	76.1	3.964 (3.2;3;9)	76.1	4.072	72.1
**6**	4.396 ?	69.2	4.43	68.9	4.307 (3.4)	70.3	4.43	68.9
**6**	-	-	4.37	-	-	-	4.37 (dd)	-

**Table 7:** Arithmetical differences for the ^1^H-NMR chemical shift of **4** and **9**, in D_2_O.

	Δ (ppm) αα		
	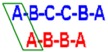	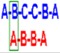	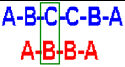
**1**	0.05	−0.06	−0.05
**2**	0.30	−0.04	−0.05
**3**	0.19	0.03	−0.11
**4**	0.08	−0.40	−0.07
**5**	0.46	−0.06	−0.05

**Table 8:** Arithmetical differences for the ^1^H-NMR chemical shift of **6** and **10**, in D_2_O.

	Δ (ppm) αβ					
	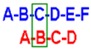	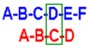	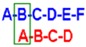	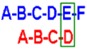	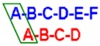	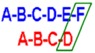
**1**	0.04	0.03	0.02	0.01	<0.01	0.12
**2**	0.02	−0.01	0.06	0.04	0.31	0.34
**3**	0.04	−0.04	0.10	0.05	0.34	0.28
**4**	−0.02	−0.02	−0.30	−0.32	0.16	0.12
**5**	0.01	−0.04	0.02	−0.04	0.16	0.18

In general, the chemical shift values for analogous positions of corresponding rings are comparable. In the case of α,αand α,βdiastereoisomers, internal rings bearing the C-C bond show very similar *δ* values, indicating that their chemical properties are not influenced by the length of the chain. As to the rings vicinal to the C-C linked ones, it has to be noted that they are external rings in the case of maltose dimers (rings A and D), while they are internal rings in the case of maltotriose dimers (rings B and E). Therefore, ring A of **9** has been compared both with rings A and B of the **4** observing that, ring A of the maltose dimer appears to be more similar to the internal ring B rather than the external one A of the maltotriose dimer, aside from position 4 that is involved in different bonds. Similarly, taking into consideration the α,β conformation, *δ* values of external rings A and D of the maltose dimer are respectively similar to the rings B and E of the maltotriose dimer. A plausible explanation for such behaviour could be that the C-C bond acts as an element of rigidity, thus affecting the mobility of the rings, where the number of rotational degrees of freedom is proportional to the distance from the C-C bond. As a consequence, chemical shift values for the maltose dimers’ external rings are influenced by the presence of the C-C bond on the vicinal ring and behave likewise the internal ones B and E of the corresponding maltotriose dimers. The influence of the C-C bond was confirmed by obtaining the ^1^H-NMR spectrum of **10** and **6** at different temperatures (303 K, 328 K). The change in the chemical shift of the ^1^H-NMR resonances of H-1B and H-1C of **10** and **6**, respectively, is less pronounced than that of anomeric protons of the external rings (Supplementary Table S1).

## 3. Experimental

### 3.1. General

Maltotriose, maltose and solvents were purchased from Sigma-Aldrich and used as received. Acetonitrile was distilled under nitrogen atmosphere over CaH_2_ before using for electrolysis reaction. Analytical TLC were performed on silica gel pre-coated on aluminum plates and compounds were detected by staining with 4% concentrated sulfuric acid in MeOH/H_2_O 1:1 v/v. Separation of maltotriose dimer diastereoisomers was performed with semi-preparative HPLC system equipped with pump (Smartline 1000 pump, Knauer), auto sampler (HT310L METROLAB), semi-preparative column and pre-column filled with the same stationary phase (UniverSil C18 250 mm × 10 mm, particle size 5 μm), 6-Port/2-Channel Switching Valve (Knauer), Refractive Index detector 2300/2400, Knauer). The mobile phase acetonitrile-water, 55:45 v:v was thermostated at 28 °C. It was isocratically eluted at a flow-rate of 5.0 mL/min and injection volume was 250 μL. The effluent was monitored by Refractive Index detector and peak fractions were collected according to the elution profile.

### 3.2. General Procedure for the Synthesis of Sulfated Maltotriose C-C Linked Dimers ***4**–**6*** and Sulfated Maltose C-C Linked Dimers ***9**,**10***

**Figure 6:**
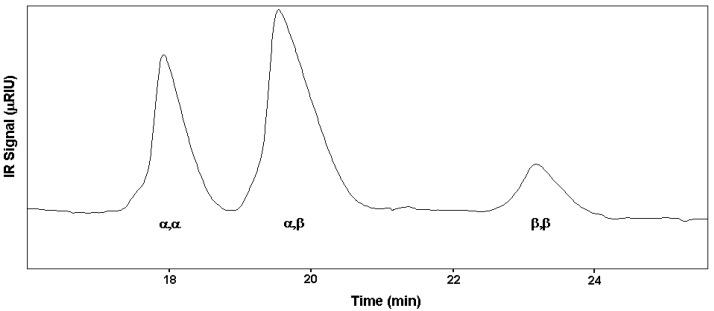
HPLC chromatogram of acetylated maltotriose dimers mixture.

Acetylated maltotriose dimers mixture was obtained from acetobromomaltotriose according to a literature procedure [25]. Their mixture was partially fractionated by flash chromatography on silica gel using a gradient from hexane-ethyl acetate 20:80 to 100% acetyl acetate as mobile phase and evaporated to dryness under reduced pressure. Fractions enriched in each diastereoisomer were further purified by means of semi-preparative HPLC. Around 120 mg of enriched fraction were dissolved with appropriate HPLC mobile phase (acetonitrile–water 55:45 v/v) at a concentration of 12.5 mg/mL and fractionated at flow rate of 5 mL/min (130 MPa system pressure) by 40 injections (250 μL injected volume x each injection). Figure 6 allows appreciating the dimers mixture separation efficiency by UniverSil (Fortis) C18 250 mm × 10 mm. Detector: Refractive Index.

Every pure diastereoisomer was deacetylated and sulfated. Sulfation was performed in pyridine by means of SO_3_–Py complex (0.75 M, 10 equiv./equiv. OH^−^) at 80 °C in a flask sealed with CaCl_2_ pipe. The reaction was stopped after 6 h, cooled to room temperature and then neutralized with a saturated NaHCO_3_ solution (about 5 mL) and finally dried. To remove the salts in excess, the crude reaction mixture was dissolved in water and loaded on TSK-gel HW40S column (5 × 100 cm) using water/EtOH 9:1 v/v as eluent with a flow rate of 5 mL/min. Collection time for each test tube was 1.2 min. The absorbance of test tubes was evaluated at 210 nm obtaining an UV profile, used to identify desalted sample. The fractions corresponding to the sample were collected together, dried and then lyophilised to afford the sulfated compound as a white powder. Sulfation reaction was repeated three times affording **4**–**6** products. The product of every sulfation step was isolated and characterised by NMR. The preparation of **9**,**10** followed quite the same procedure. In this case HPLC purification was not necessary, as flash chromatography was sufficient to isolate pure intermediate compounds.

### 3.3. NMR Characterisation of Sulfated Maltotriose C-C Linked Dimers ***4**–**6*** and Sulfated Maltose C-C Linked Dimers ***9**,**10***

^1^H-NMR and ^13^C-NMR spectra were recorded at 303 K with a Bruker Avance 500 or Avance 600 spectrometers both equipped with 5 mm TCI cryoprobe and with a Bruker AMX 400 MHx operating at 100 MHz for ^13^C measurements. Samples were dissolved in deuterium oxide. In order to reduce the water amount, the samples were exchanged three times with high-purity D_2_O (99.996%) and finally dissolved in 0.6 mL of the same solvent. Chemical shift are expressed in ppm respect to external deuterated TSP. Assignments of the NMR spectra of α,α, α,β and β,β diastereoisomers were made by means of ^1^H-NMR, COSY, TOCSY, HSQC, DEPT-HSQC and HMBC experiments. One-dimensional ^1^H-NMR spectroscopy was performed on all the protected, deacetylated and sulfated purified diastereoisomers. Comparison between ^1^H-NMR spectra of reagents and products was used to check the achievement of the deacetylation and sulfation reactions. The ^1^H-COSY spectrum was used to assign the chemical shift of the glucose units of each diastereoisomers; ^1^H-TOCSY spectrum was used to confirm the obtained assignments. On the base of results obtained, HSQC and HMBC experiments were considered to assign ^13^C chemical shift for each position of different glucose units of **6**.

### 3.4. Mass Characterisation of Sulfated Maltotriose C-C Linked Dimers ***4**–**6***

The samples were analysed on a UPLC chromatograph (PLATIN blue, Knauer) interfaced to an ESI-Ion Trap mass spectrometer (AmaZon SL, Bruker Daltonics). Each sample (5 mg/mL; 10 μL of injection volume) was loaded onto a C18 column (2.0 × 100 mm, 1.8 μm, Knauer) and eluted at 0.4 mL/min performing a linear gradient from 10% to 50% in 60 min. Mobile phase solutions A and B were dibutylamine 10 mM, acetic acid 10 mM in H_2_O/CH_3_CN 90:10 (v/v) and dibutylamine 10 mM acetic acid 10 mM in ACN 100%, respectively. The column temperature was maintained at 35 °C.

The electrospray interface was set in negative ionization mode (Spray Voltage +4,200 V, Cap Exit −140 V), to record total ion current profiles in the *m/z* 300–2000 mass range. Nitrogen was used as a drying (9 L/min) and nebulising gas (30 p.s.i.) and the ion transfer capillary was held at 200 °C. LC-MS profiles and mass spectra were elaborated by DataAnalysis (Bruker Daltonics).

## 4. Conclusions

Semi-synthetic sulfated maltotriose C-C linked dimers were prepared with the aim of finding carbohydrate-based heparin mimetics. They provided significant improvements over the 1990s story of C-glycosides chemistry and applications. They came from an electrochemical unique approach able to double sugar units in one step. Following this first essential step huge efforts were devoted to the sulfation process in term of synthesis and characterisation. The results we obtained not only offer a definitely satisfying chemical and analytical picture, but can also explain the first very encouraging biological activities. Samples **4** and **6** present a high degree of sulfation, suitable for biological activities. They are non-natural hexasaccharides, long enough to have biological activities. Sample **5** is a complex mixture of differently sulfated forms of a non-natural hexasaccharide. Samples **9** and **10** present a high degree of sulfation. They are non-natural tetrasaccharides, too short to have biological activities. Compound **6** appears to be the best sample in term of biological activities, it inhibits heparanase, P-selectin and is an anti-metastatic drug for different tumours. Its preferred conformation is quite similar to that of heparin, and this can justify also its biological activities. Compound **4** inhibits heparanase, but does not inhibit P-selectin, so it is an anti-metastatic drug only for some species of tumours. Its preferred conformation differs for some extent from that of heparin, and this could be related to its biological activities. **5** has no biological activities, maybe due to negative conjunction of low sulfation degree and unsuitable conformation. 

## Supplementary Materials

Supplementary materials can be accessed at: http://www.mdpi.com/1420-3049/17/8/9912/s1.
