# Supplementary material for: Anti-metastatic Semi-synthetic Sulfated Maltotriose C-C Linked Dimers. Synthesis and Characterisation
Source: Molecules. 2012 Aug 17;17(8):9912–30. doi: 10.3390/molecules17089912 (PMC3646267; doi:10.3390/molecules17089912)
Supplement: Supplementary file 1 [file molecules-17-09912-s001.pdf]

Supplementary Materials

NMR spectra of compounds 4, 5, and 6.
NMR spectra of compound 10 at different temperatures.

SI1. NMR spectra of Compound 4.

Figure S1. ^{1}H-NMR spectrum of **4**, in D_2O. 500 MHz.

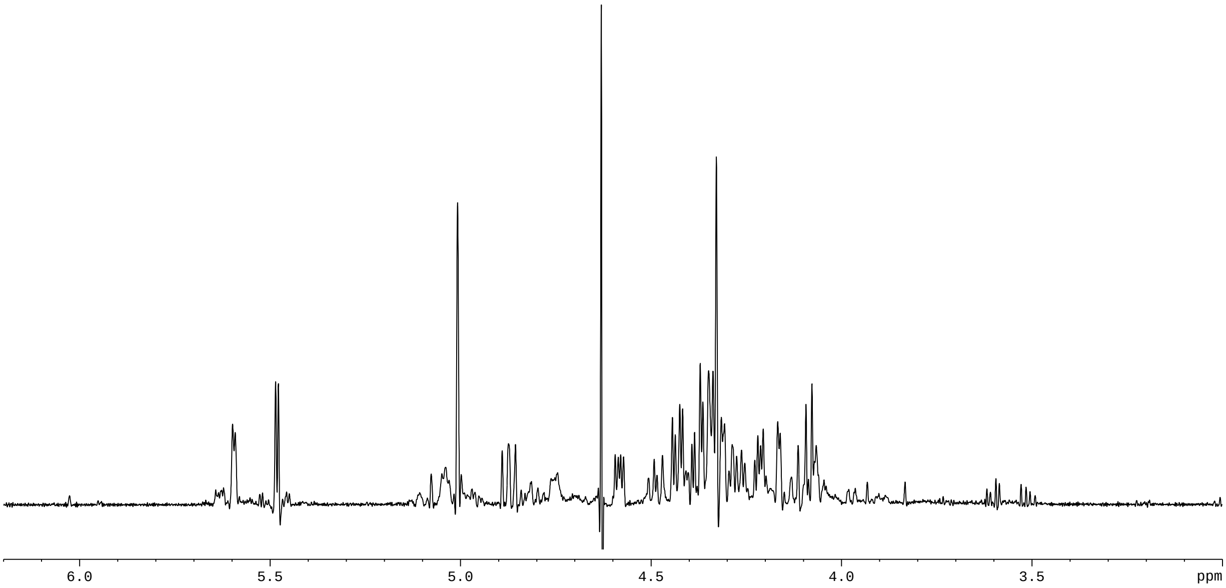

Figure S2. ^{1}H-^{13}C HSQC spectrum of **4**, in D_2O. 500 MHz.

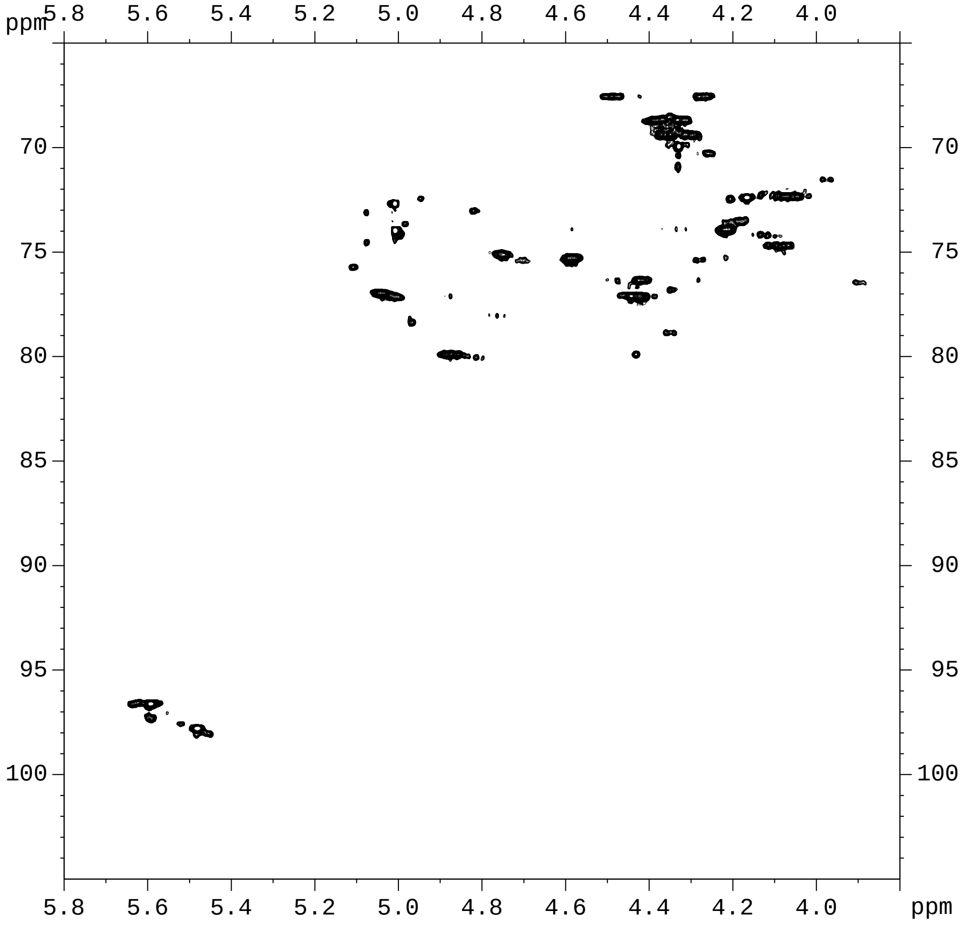

Figure S3. 2D ¹H-NMR COSY spectrum of **4**, in D_2O. 500 MHz.

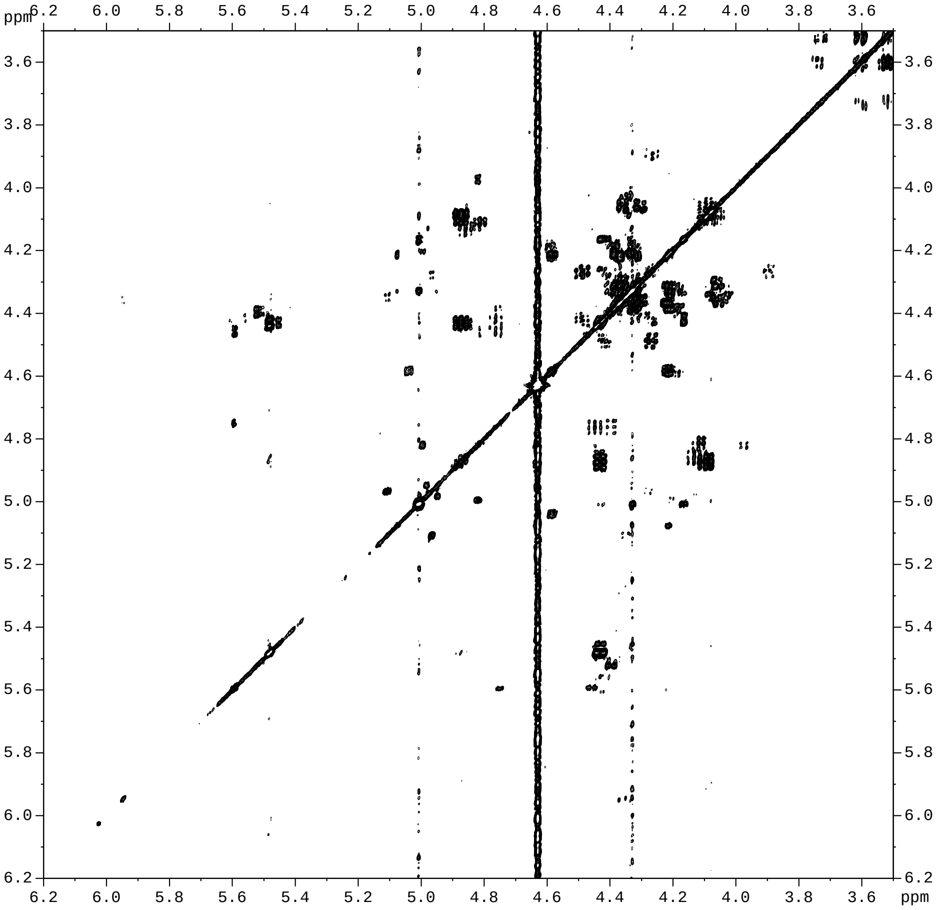

Figure S4. 2D ¹H-NMR TOCSY spectrum of **4**, in D_2O. 500 MHz.

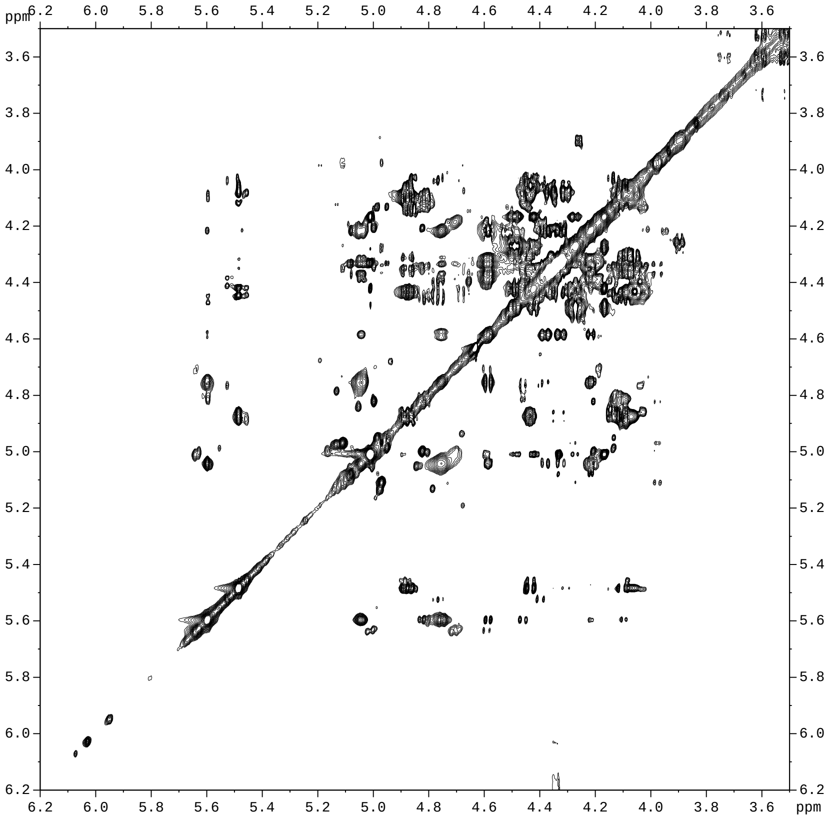

SI2. NMR spectra of Compound 5.

Figure S5. ^{1}H-NMR spectrum of **5**, in D_2O. 600 MHz.

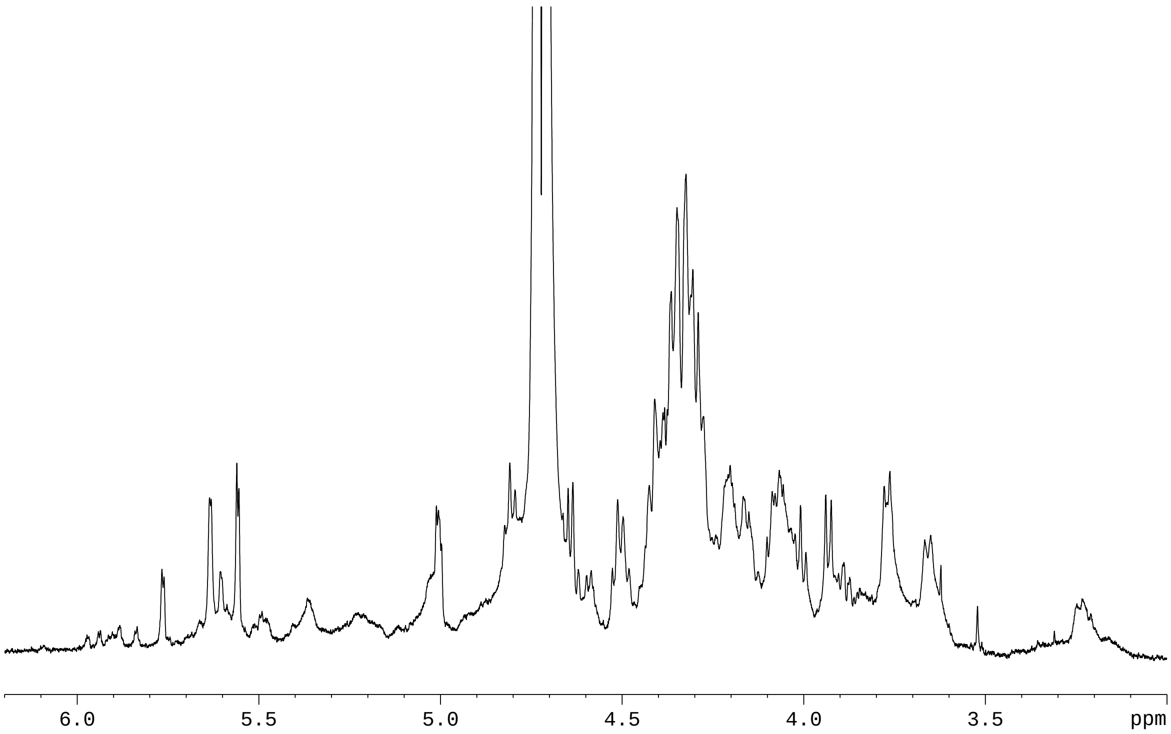

Figure S6. ^{1}H-^{13}C HSQC spectrum of **5**, in D_2O. 600 MHz.

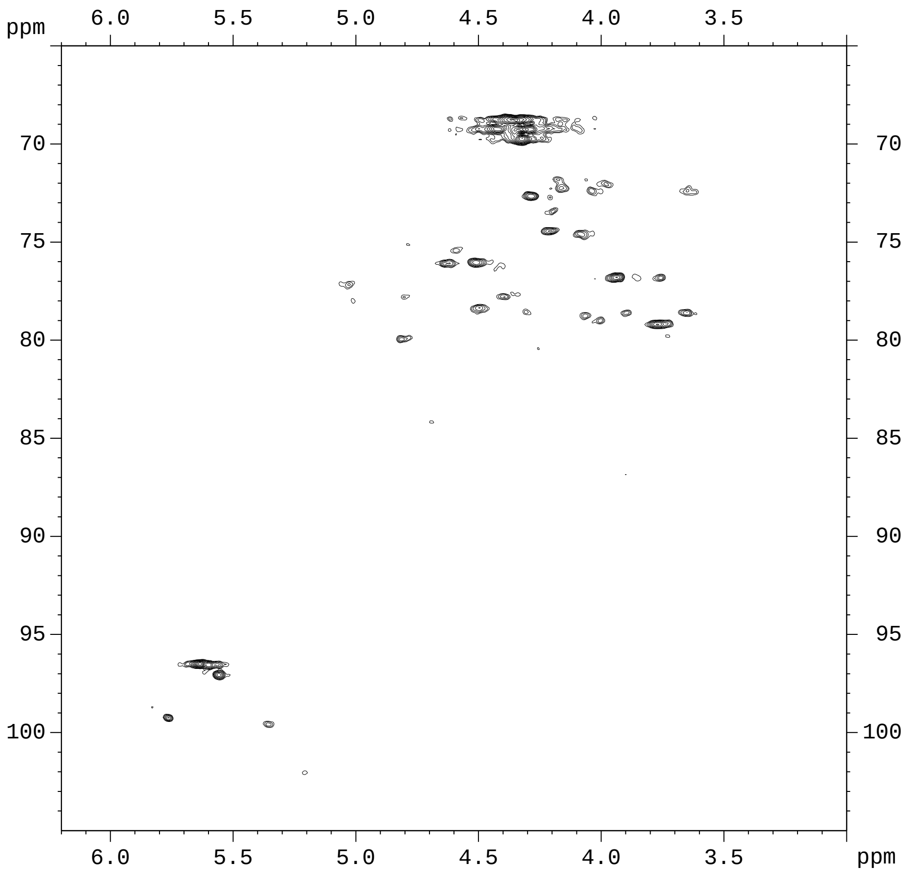

Figure S7. 2D ^{1}H-NMR COSY spectrum of **5**, in D_2O. 600 MHz.

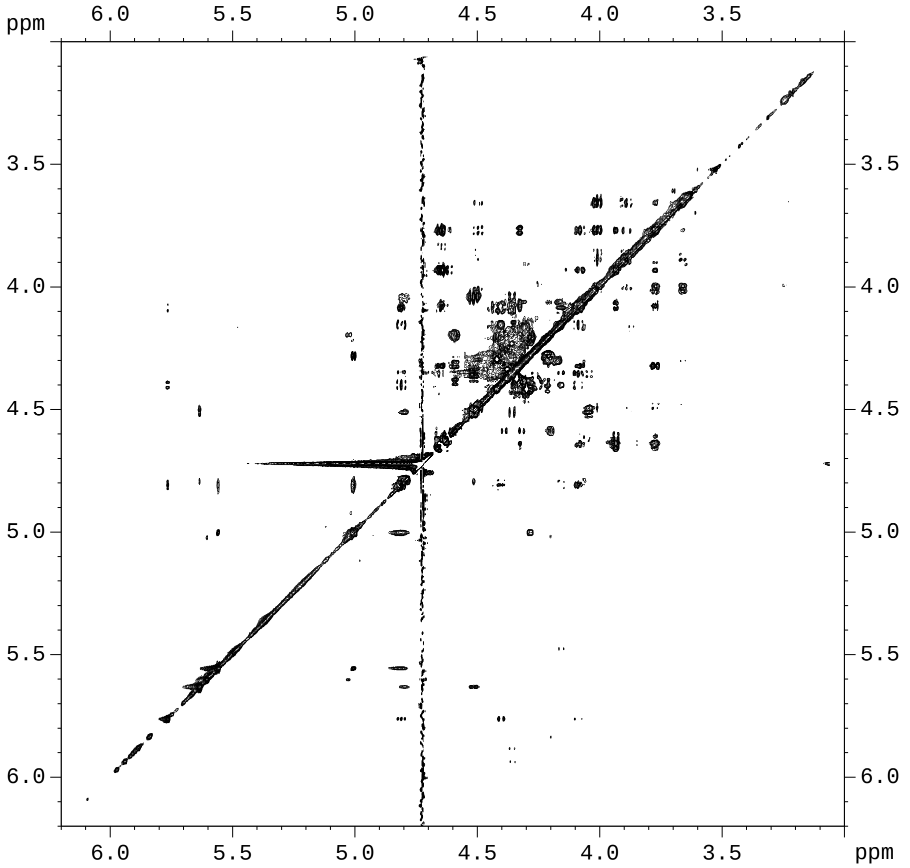

Figure S8. 2D ^{1}H-NMR TOCSY spectrum of **5**, in D_2O. 600 MHz.

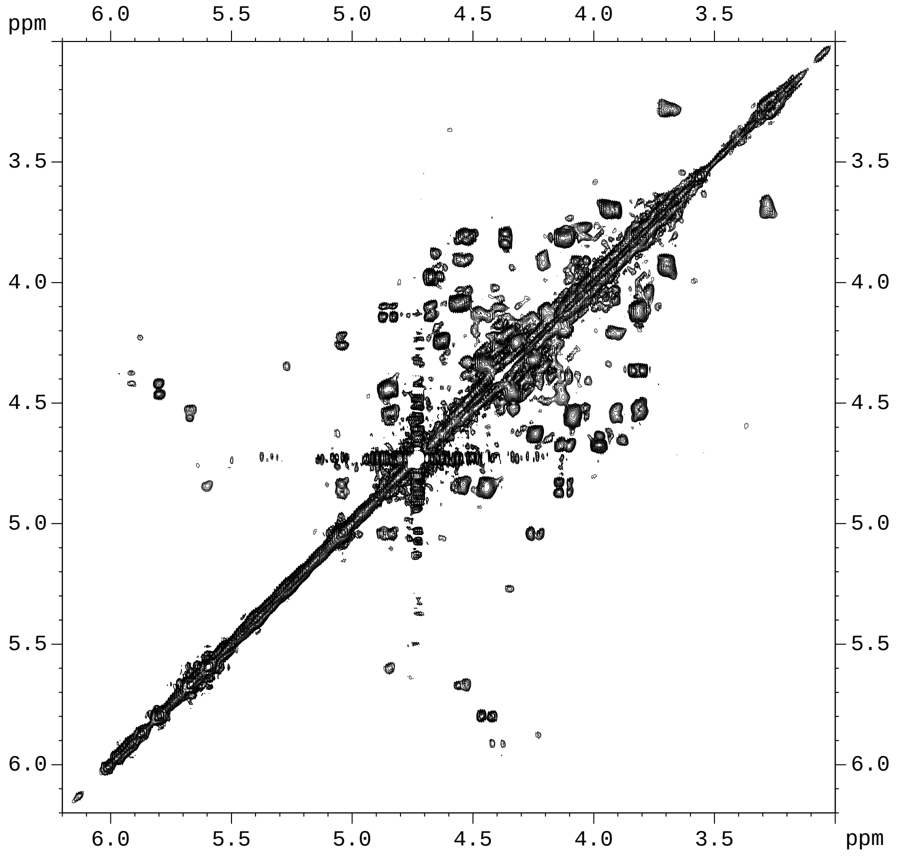

SI3. NMR spectra of Compound 6.

Figure S9. 2D ^{1}H-NMR COSY spectrum of **6**, in D_2O. 600 MHz.

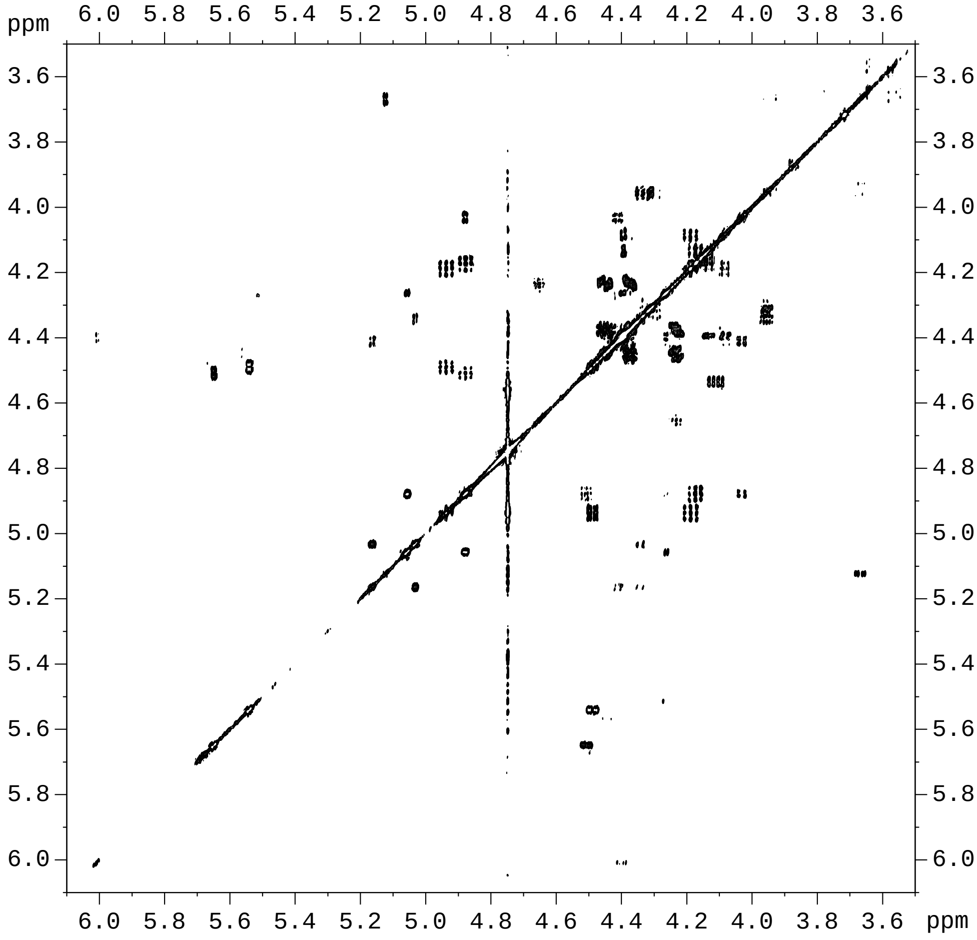

SI4. Comparison of NMR spectra of compounds 6 and 10 at different temperatures.

Figure S10. ^{1}H-NMR spectra of compound **10** in D_2O, 500 MHz, at different temperatures.

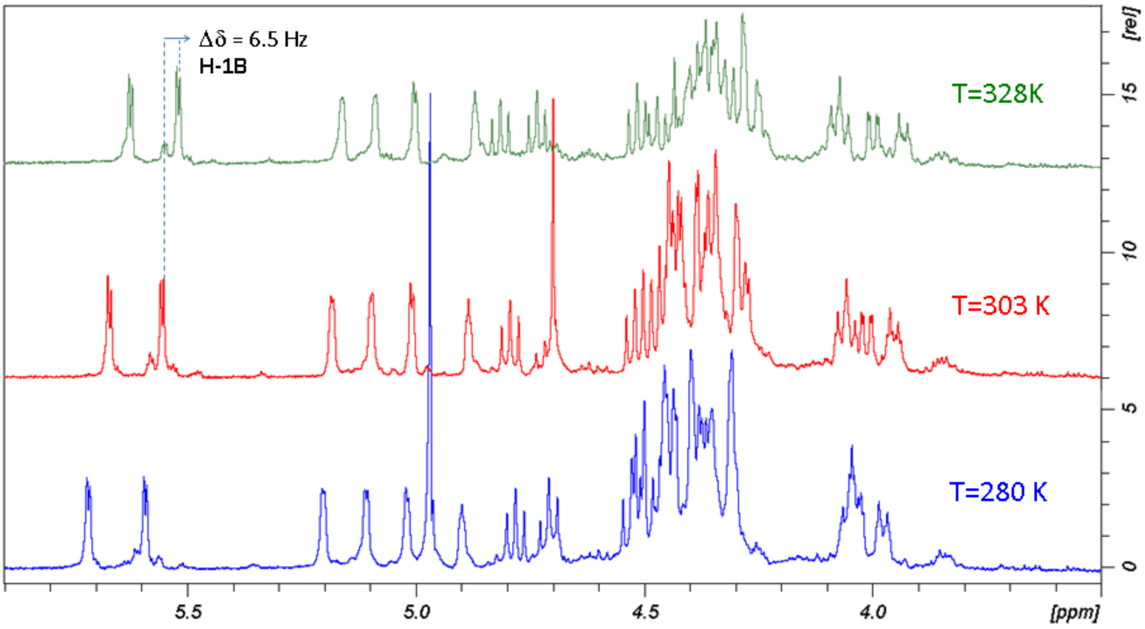

Table S1. ^{1}H-NMR chemical shift differences (in Hz) at 303 and 328 K for the anomeric protons of compounds **10** and **6**. D_2O, 500 MHz.

**Hexamer Ring**	**A**	**B**	**C**	**D**	**E**	**F**
Δδ	**20-23**	**17**	**7.5**	**nd**	**13**	**20-23**
**Tetramer Ring**		**A**	**B**	**C**	**D**	
Δδ		**22**	**6.5**	**nd**	**17**	
